# Cancer Vaccine Targeting Mutated GNAQ-Expressing Uveal Melanoma

**DOI:** 10.3390/cancers18030480

**Published:** 2026-01-31

**Authors:** Vitali Alexeev, Mizue Terai, Sergei Koshkin, Olga Igoucheva, Takami Sato

**Affiliations:** 1 Department of Medical Oncology, Sidney Kimmel Cancer Center, Thomas Jefferson University, 1015 Walnut Street, Philadelphia, PA 19107, USA; mizue.terai@jefferson.edu (M.T.); sergei.koshkin@jefferson.edu (S.K.); takami.sato@jefferson.edu (T.S.); 2 Department of Dermatology and Cutaneous Biology, Sidney Kimmel Medical College, Thomas Jefferson University, 233 S. 10th Street, Philadelphia, PA 19107, USA

**Keywords:** DNA vaccine, uveal melanoma, fusion antigen, VP22, PADRE, protective immunity, mutated GNAQ

## Abstract

**Background/Objectives:** Uveal melanoma (UM) is the most common intraocular malignancy in adults. Although brachytherapy of the primary tumor provides an approximate 80% five-year survival, with time, nearly half of patients experience predominant liver metastases. It was proposed that malignant cells migrate early and stay dormant as they adapt to the liver microenvironment. We propose that cancer vaccine-mediated activation of UM-targeted immunity in primary UM patients could prevent progression of metastatic disease from dormant cells or malignant seeds. Thus, this study explored DNA vaccination as a measure to educate the immune system to recognize the most common UM-associated Q209L tumor driver mutation in GNAQ and GNA11 G-alpha proteins. **Methods:** Several DNA constructs encoding mutated GNAQ were developed and tested for activation of UM-reactive T cells in HLA-A2/Hd transgenic mice and human T cells ex vivo. **Results:** Constructs containing immune-enhancing PADRE and VP22-derived epitopes boosted T cell responses against mutant GNAQ, which correlated with reduced experimental lung metastases. Ex vivo dendritic cell-mediated T cell activation with vaccine constructs containing optimized structure produced cytolytic T cells that secreted IFN gamma and killed mutated GNAQ-expressing UM cells in vitro. **Conclusions:** These findings propose the utility of the fusion DNA vaccines in eliciting T cell immunity against UM cells bearing the Q209L mutation in GNAQ/GNA11 protein to prevent the establishment and progression of metastatic disease.

## Simple Summary

Most effective immunotherapeutics rely on activation of a patient’s immune system to fight cancer; however, they are only somewhat effective against metastatic uveal melanoma (MUM). This study explored DNA vaccination as a way to teach the immune system to recognize tumors and prevent the progression of metastatic lesions from dormant malignant cells and tumor seeds. Our findings indicate that cancer-fighting immune cells activated by DNA vaccines in animals and human cells can recognize and attack tumor cells characterized by the common UM-associated cancer-driving mutation. An enhanced form of this vaccine containing multiple parts and improved structure was even more effective in activating cancer-fighting immune cells. These data suggest that such a vaccine could be further improved toward the design of a novel MUM-preventive strategy.

## 1. Introduction

Uveal melanoma (UM) is the most common intraocular malignancy in adults, accounting for 3.1% of all recorded cases of melanoma [1,2]. Although treatment of the primary intraocular lesions provides a favorable outcome for most of the patients [3], up to 50% of patients subsequently develop metastatic UM (MUM), most commonly in the liver, and succumb to the disease [4] even with the most advanced immunotherapies. The overall response rate in immune checkpoint inhibitors (ICIs) was low (3–18%), and median overall survival (OS) and progression-free survival (PFS) were reported to be 12–16 months and 2.7–3.0 months, respectively. Objective response rate to a novel T cell engager, Tebentafusp, was also low (9%) with a median PFS of 3.3 months and a median OS of 21.7 months in treatment-naïve MUM patients [5,6,7]. Low response to ICI and Tebentafusp therapies could be caused by the lack or paucity of the naturally occurring MUM-recognizing T cells and by the lack of T cell recruitment to MUM and quiescent/exhausted phenotype of the intratumoral T cells. These findings suggest that new modalities are needed to activate UM-specific T cell response or educate the immune system to recognize UM-associated antigens. Most primary UM and MUM share a tumor driver activating point mutation in highly homologous GNAQ and GNA11 Gα proteins, a substitution of glutamine at position 209 with leucine (Q209L) [8,9]. In our clinic, 62.5% of MUM cases are characterized by Q209L mutation in Gα proteins [10]. Given that branched amino acids, such as leucine, enhance binding of the peptides to HLA-A*02:01, we aimed this proof-of-concept study at evaluating immunogenicity of the Q209L-harboring GNAQ and at the potential to elicit T cell-mediated immunity using the deoxyribonucleic acid (DNA) vaccine platform. Multiple clinical studies highlight the advantages of DNA vaccines over other cancer vaccine platforms (reviewed in [11]). They elicit systemic, antigen-specific immune responses and durable immunologic memory while remaining safe, well-tolerated, and suitable for repeated administration. Unlike viral vaccines, they pose no risk of pathogenic infection or neutralizing antibody induction, and unlike RNA vaccines, they are stable and easily scalable. Moreover, DNA vaccines could be customized using standard molecular techniques for personalized therapies. Combining different immune-enhancing epitopes and mutated GNAQ (mtGNAQ), we optimized vaccine composition and assessed vaccine-mediated activation of UM-recognizing T cells ex vivo and in vivo. Despite the limitations (listed in the Section 4), our findings support mtGNAQ as a promising target for tumor-directed immunity.

## 2. Materials and Methods

### 2.1. DNA Vaccine Construction and Vaccination

**Figure 1:**
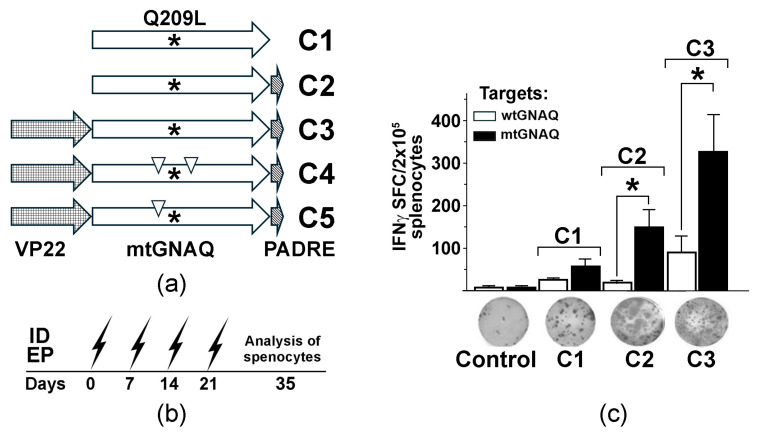
Design and testing of mtGNAQ DNA vaccine-induced T cell activation in vivo. (**a**) Diagram illustrating designs of the fusion vaccines containing VP22 and mtGNAQ ORFs and PADRE. Asterisks and down triangles indicate the position of the Q209L mutation and anchoring substitutions, respectively. (**b**) Timeline of DNA vaccinations via intradermal electroporation (ID EP, zigzag arrows) and collection of the splenocytes. (**c**) Column chart illustrating enumeration of IFNγ spot forming cells in differently vaccinated mice. Data are presented as an average of five independent measurements ± SD. Images of representative ELISpot wells corresponding to respective treatments are shown below the columns. In panels, statistical significance (*p* < 0.05) is indicated by asterisk.

To generate the mtGNAQ-specific DNA vaccine, full-length mouse GNAQ cDNA was amplified from total RNA isolated from Melan-a mouse melanocytes [12] using the Superscript III RT kit (Thermo Fisher, Waltham, MA, USA, #18080051) and PfuUltra II DNA polymerase (Agilent, Santa Clara, CA, USA, # 600670). There is 99.9% homology between mouse and human GNAQ protein sequences with exception of the hydrophobic alanine (A171) in human GNAQ that changes to polar serine in the mouse protein (Figure S1); therefore, a DNA vaccine targeting Q209L mutation could be used in both humans and mice. The cDNA was ligated into the pEF6/V5-His TOPO vector (Thermo Fisher, # K961020). Q209L and anchoring substitutions were introduced into the cDNA using a Phusion site-directed mutagenesis system (Thermo Fisher, #F541). Four vaccine vectors containing full-length wild-type and mutant GNAQ (mtGNAQ) cDNA alone or fused in-frame with PADRE (mtGNAQ-PADRE) with and without VP22 cDNA at the 5′ end (VP22-mtGNAQ-PADRE) were constructed (Figure 1a) using standard molecular cloning techniques. PADRE- and VP22-encoding sequences have been used to enhance the activation of T helper cells and cytotoxic T cells [13,14]. In vivo DNA vaccination was performed using the BTX ECM 830 system as described in our prior studies [15].

### 2.2. Cells

HLA-A*02:01-positive human periferal blood mononucleated cells (PBMC) were isolated from human whole periferal blood (StemCell Technologies, Vancouver, BC, Canada, Leukopack, Cat#200-0043). Primary monocytes and T cells were isolated from PBMC by negative selection using cell type-specific negative selection kits (StemCell Technologies, Cat# 19359 (CD14+ monocytes) and Cat#19359 17961 (naïve pan-T cells)).

HLA-A*02:01-positive UM002 cells were isolated in the laboratory from uveal melanoma metastasis; 92.1 cell line was purchased from Millipore Sigma (Cat.# 13012458, Burlington, MA, USA); primary human melanocytes 1801c were kindly provided by Dr. Zalfa Abdel-Malek (University of Cincinnati, Cincinnati, OH, USA). CM006 cutaneous melanoma cells were isolated from cutaneous melanoma metastasis in the laboratory.

### 2.3. IFNγ ELISpot Assay

Mouse and human T cell activation was evaluated by IFNγ ELISpot assays using species-specific kits (eBioscience Santa Clara, CA, USA, #88-7384-88; ImmunoSpot, Rutesheim, Germany, #hIFNg), as devised by the manufacturers. Mouse or human melanocytic cells expressing mutated or wild-type GNAQ were used as targets.

### 2.4. mtGNAQ Model for In Vivo Animal Studies

To generate a suitable mouse cell model, spontaneously immortalized non-tumorigenic mouse melanocytes, Melan-a, were stably transduced with expression vectors encoding HLA-A2/K^b^ (Addgene, Watertown, MA, USA, #14906, a gift from L. Sherman [16]) and full-length mtGNAQ via nucleofection. After selection, the cells acquired TPA-independent growth in vitro and the ability to produce blue nevus-like lesions in the skin (Figure S2). The resultant cells were used in all animal experiments. All animal studies were approved by the Institutional Animal Care and Use Committee. To establish pulmonary metastases, mtGNAQ-expressing cells (5 × 10^5^ cells per mouse) were injected into HLA-A2/Hd transgenic mice via tail vein. Once lesions were established (2 weeks), animals were vaccinated via intradermal (ID) electroporation (EP), as described previously [15]. Five mice per cohort were used in all experiments.

### 2.5. Ex Vivo T Cell Activation and Analysis

Ex vivo activation of HLA-A*0201-positive T cells with autologous dendritic cells (DCs) was performed as previously described [17], with modifications. Briefly, CD14^+^ monocytes and CD3^+^ pan-T cells were isolated from PBMC of healthy donors. Monocytes were differentiated into DCs using a DC differentiation kit (StemCell Technologies), and immature DCs were transduced with DNA vaccines via nucleofection (#VPA-1004; Lonza, Basel, Switzerland). DC maturation was induced by adding 50 ng/mL TNFα, and DCs were exposed to autologous T cells at DC:T ratio of 1:40. After 24 h, DC maturation markers CD80 and CD86 as well as vaccine products were assessed by fluorescence activated cell sorting (FACS) and Western blot, respectively (Figure S2). After the initial activation and two re-stimulations with vaccine-transduced DCs, pooled polyclonal T cells were exposed to wtGNAQ and mtGNAQ targets. T cell activation was assessed using the IFNγ ELISpot assays. DCs and T cells from the three donors were tested. All kits, reagents, and culture media used for DC and T cell isolation, preparation, and culture were purchased from StemCell Technologies (Supplementary Materials S1). The cytotoxic activity of activated T cells was assessed using a FACS-based cell toxicity assay (Millipore, Burlington, MA, USA, #4500-0230) and the GuavaEasyCyte FACS system. Data were analyzed using GuavaSoft 2.7 software.

### 2.6. Immuno-Sequencing and Analysis of T Cell Repertoire

Evaluation and analysis of the T cell repertoire of the UM-recognizing vaccine-activated T cells were performed on RNA samples isolated from activated T cells using services provided by Creative Biolabs (Shirley, NY, USA).

### 2.7. Statistical Analysis

One-way analysis of variance (ANOVA) and paired *t*-test were performed using the GraphPad Prism 10.1.1 software. All experiments, except for RNA sequencing, were performed with at least three biological replicates. Statistical significance was set at *p* < 0.05.

## 3. Results

### 3.1. Fusion DNA Vaccine Enhance Q209L-Specific T Cell Immunity In Vivo

To evaluate whether the presence of the mutant leucine in Q209L enhances presentation of the mutated peptides by the MHC molecules, we completed an in silico analysis of the Q209L-harboring peptides (Table S1). This analysis predicts that the presence of mutant L in P7, P9, and P8 positions of the nonamers increases the binding of the peptides to HLA-A*01:01, A*02:01 and A*03:01, respectively, converting these peptides to potential weak binders (Table S1).

To assess the immunogenicity of mtGNAQ, we created three DNA vaccine constructs encoding mutated GNAQ (mtGNAQ) (GNAQ are highly conservative among species. Amino acid sequences of mouse and human GNAQ are 99.9% identical with only one amino acid difference (A/S) at position 170 (Figure S1)) alone (construct C1) or fused in-frame with Pan DR-binding epitope (PADRE) on 3′ end (construct C2) and herpes simplex virus VP22 encoding sequence on 5′ end of mtGNAQ (construct C3) (Figure 1a). To define whether these constructs could activate mtGNAQ-specific immunity, naïve HLA-A2/Hd mice were vaccinated via intradermal (ID) electroporation (EP). This method provides efficient vaccine expression in mouse skin (Figure S2a). Vaccine administration sites were pre-treated with CCL21. Our previous studies demonstrated that such pre-treatment significantly enhances recruitment leukocytes, including CCR7^+^ dendritic cells and T cells, and improves DNA vaccine efficacy [15]. Two weeks after four consecutive once-a-week vaccinations (Figure 1b), the activation of mtGNAQ-specific T cells was assessed by IFNγ ELISpot assay. Melan-a cells engineered to express mtGNAQ and HLA-A2/Kb hybrid MHC (MelanQ-A2) (Figure S2b–e) were used as targets. When injected intradermally into mouse skin, these cells produce blue nevus-like lesions (Figure S2f). Parental Melan-a mouse melanocytic cells [12] were used as control. This assay showed that vaccination with mtGNAQ alone (construct C1) produced a small number of IFNγ spot-forming cells (SFCs). The fusion of mtGNAQ with PADRE (construct C2) led to a marginal increase in SFCs, whereas VP22-mtGNAQ-PADRE (construct C3) provided the highest number of activated T cells when exposed to mtGNAQ targets (Figure 1c). Insignificant T cell activation after exposure to Melan-a cells suggested that T cells from C3-vaccinated mice specifically recognize targets expressing mtGNAQ.

### 3.2. Fusion DNA Vaccine Potentiates Cellular Immunity Sufficient to Prevent Metastatic Lesions

**Figure 2:**
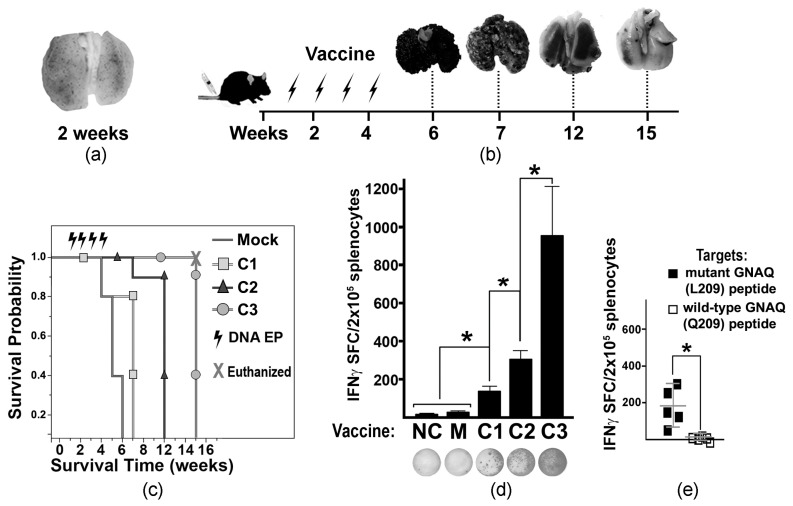
Tumor progression and vaccine-mediated T cell activation in tumor-bearing mice. (**a**) A2. cells. (**b**) Diagram illustrating the experimental timeline of vaccine administration and collection of tumor-affected lungs (representative images are shown above the timeline). Arrows indicate the times of ID EP. (**c**) Kaplan–Meier viability plot illustrating survival probability of tumor-bearing mice (weeks) vaccinated with different DNA constructs (as indicated in the key). Mice in the C3-vaccinated cohort were euthanized at week 15 to terminate the experiment with hazard ratio (HR) = 0.04 compared to the Mock-treated cohort. (**d**) Column chart illustrating the IFNγ ELISpot assay defining T cell activation in differently vaccinated mice. MelanQ-A2 cells were used at targets. The DNA vaccine constructs (C1, C2, and C3) are indicated below the columns. NC—negative control (splenocytes from naïve non-tumor-bearing mice); M—Mock-treated tumor-bearing mice. Data are presented as average SFCs from splenocytes collected from four animals per cohort ± SD. Representative photographs of ELISpot wells are shown below respective columns. (**e**) Scatter plot chart illustrates the specificity of T cell activation (IFNγ ELISpot assay) toward mutant GNAQ peptides loaded onto human T2 (HLA-A*0201) cells. The averages ± SD are shown in the charts. Splenocytes isolated from the C3-vaccinated mice were used. Statistical significance (*p* < 0.05) is indicated by an asterisk.

To develop a MUM-like experimental metastatic model, tumor-like cells (MelanoQ-A2) were inoculated intravenously (iv) into HLA-A2/Hd mice. Formation of multiple small pulmonary lesions confirmed the suitability of this cell model (Figure 2a). Then, experimental and control mice were iv inoculated with MelanQ-A2 cells and vaccinated via ID EP with Mock and mtGNAQ-encoding constructs four times once a week. Mock-, C1-, and C2-vaccinated animals develop respiratory distress within 6, 7, and 12 weeks after tumor inoculation, respectively. C3-vaccinated mice did not show signs of pulmonary distress for 15 weeks (Figure 2b). Evaluation of lung metastases showed that vaccination with C3 construct substantially reduced progression of pulmonary lesions (Figure 2b) and enhanced survival of the tumor-bearing mice beyond 7 weeks (Figure 2c). Reduction of tumor burden coincided with T cell activation quantified by IFNγ ELISpot (Figure 2d). Splenocytes from C3-vaccinated mice showed a significantly higher number of IFNγ SFCs than splenocytes from other vaccinated and control cohorts (Figure 2d). Considering that HLA-A2/Hd mice can generate T cells that recognize HLA-A2-bound epitopes [18], HLA-A2-restricted T cell response was differentiated using IFNγ ELISpot assay by exposure of splenocytes to mutated peptides bound to human T2 (HLA-A*0201^+^) cells. It showed the presence of T cells recognizing HLA-A*02:01-bound mutant GNAQ peptides (Figure 2e).

### 3.3. Anchor Modifications of mtGNAQ Enhance Human T Cell Activation Ex Vivo

**Figure 3:**
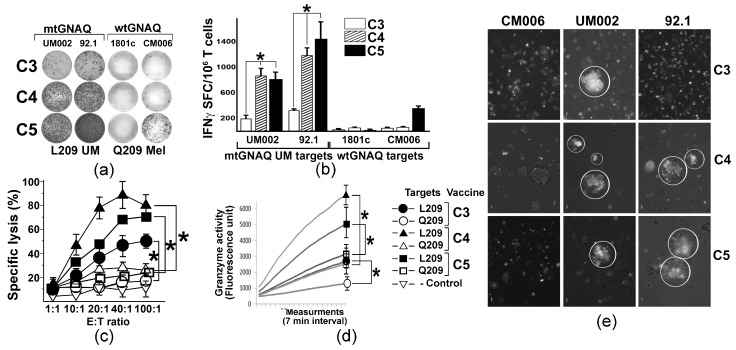
T cell activation by unmodified and anchor-modified DNA vaccines ex vivo. (**a**) Representative images illustrating the presence of IFNγ SFC in human T cells activated by different vaccine constructs (shown to the left) ex vivo; mtGNAQ-expressing UM cells and wtGNAQ-expressing melanocytic cells (indicated above the columns) were used as targets. (**b**) Column charts illustrating quantitation of IFNγ positive spots produced by T cells activated by different constructs (indicated in the key). Mutant and wild-type mtGNAQ targets are shown below the columns. Data are presented as averages of IFNγ SFC collected from three independent experiments ± SD. Assays were conducted in technical duplicates. (**c**,**d**) Charts illustrating cytotoxicity (**c**) and granzyme B activity (**d**) of vaccine-activated T cells (constructs indicated in the key to the right of the charts). In (**c**), Effector/Target (E:T) ratio is shown on the *x*-axis; specific lysis of target cells is shown on the *y*-axis. Data are collected from three independent experiments with technical duplicates and presented as an average ± SD. In (**d**), representative Granzyme B activity was measured in lysates of T cells exposed to wild-type and mutant GNAQ targets for 2 h. Activity was measured for 2 h with 7 min intervals using fluorescence-based Granzyme B activity assay. SDs at the endpoint were calculated from three independent measurements. CM006 and 92.1 cells were used as wtGNAQ and mtGNAQ targets, respectively. (**e**) Representative micrograph illustrating aggregation of Vybrant DiO-labeled ex vivo activated human T cells (white) on clusters of mtGNAQ UM cell (nonfluorescent; 92.1 and UM002) and lack of aggregation on the wild-type GNAQ-expressing cells (unlabeled, CM006). C3, C4, and C5 T cell-activating vaccine constructs are indicated to the right of the panels. Scale bar—10 µm. In all panels, statistical significance (*p* < 0.05) is indicated by asterisks.

To evaluate whether mtGNAQ-encoding fusion antigens could potentiate human T cell-mediated immunity toward mtGNAQ, we established a DNA vaccine-based ex vivo T cell activation protocol using donor-derived autologous DC and T cells. With the goal to improve mtGNAQ peptide-HLA-A*02:01 binding, predicted R202L or D205L and E212V anchoring substitutions (Table S1) were introduced into the mtGNAQ cDNA of the C3 construct. The resultant vectors were designated as C4 (R202L) and C5 (D205L and E212V) (Table S1, Figure 1a). CD14^+^ monocytes from two different donors were differentiated into immature DC (Figure S3a,b) and nucleofected with C3 and anchor-modified constructs. Then DC maturation was induced and the expression of the fusion construct was validated by Western blot (Figure S3c). Vaccine-traduced mature CD80^+^ CD86^+^ DC (Figure S3e) were exposed to autologous pan-T cells. After the initial T cell priming, two re-stimulations with vaccine-transduced DC and low-dose IL-2 were performed at 1-week intervals. Then, T cell activation was evaluated by IFNγ ELISpot assay using UM-derived mtGNAQ and control, wild-type GNAQ-expressing melanocytic cells as targets. Quantitation of SFCs showed that C4 and C5 constructs provided, on average, five times greater T cell activation than the C3 construct (Figure 3a,b). T cell activity toward wild-type GNAQ-expressing melanocytes (1801c) and cutaneous melanoma cells (CM006) was not detected, although some response to one of the tested targets (CM006) was seen in C5-activated T cells (Figure 3a,b). FACS-based cytotoxicity assay showed that all three constructs produced specific cytolytic T cells recognizing mtGNAQ-expressing UM cells (92.1). The cytolytic activity of C5-activated T cells was the greatest, although there was no significant difference between CTL activity elicited by C5 and C4 constructs (Figure 3c). CTL activity toward UM was also confirmed by the xCELLigence real-time cytotoxicity assay, which showed progressive elimination of UM targets by differently activated T cells within 2 and 5 h (Figure S3e). Significantly higher Granzyme B activity of the C5 and C4-activaed T cells confirmed a greater response toward mtGNAQ UM targets (Figure 3d). Aggregation of DiO-labeled C5 and C4 ex vivo activated T cells on clusters of mtGNAQ UM targets within 1 h and the lack of accumulation of these T cells on control targets (CM006) also support the specificity of the T cell response toward mtGNAQ-expressing cells (Figure 3e).

### 3.4. TCR Repertoire Suggests a Predominant Expansion of a Specific T Cell Clones in DNA Vaccine-Activated T Cell Population

**Figure 4:**
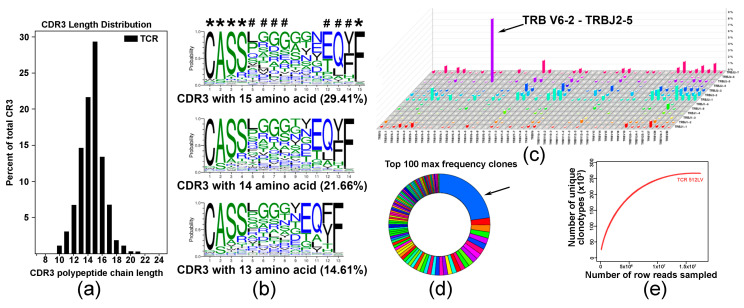
Analysis of T cell repertoire in C4-activated T cells. (**a**) Analysis of CDR3 length distribution in C4-activated T cell sample. Data are presented as percent of specific polypeptide chain length. (**b**) Analysis of amino acid conservation of the top three sequences with the largest CDR3 length distribution. Five fully conserved and three partly conserved residues are denoted with asterisk and octothorp, respectively. (**c**) V-J pairing usage analysis in TCR assembly showing preferential TRB V6-2 and J2-5 pairing in a pool of vaccine-activated T cells. (**d**) Diagram illustrating TRB distribution statistics showing that more than 30% of vaccine-activated T cells express specific TCRB (arrow). (**e**) Saturation evaluation showing that the amount of sequencing data used was sufficient and reached saturation.

To explore the diversity of the TCR repertoire in a pool of C5-activated T cells, we employed an RNA-based immuno-sequencing platform. The complementarity-determining region 3 (CDR3) length distribution (Figure 4a) and multiple sequence alignment indicated on shared CDR3 sequences with five fully conserved and three partly conserved residues are denoted with asterisk and octothorp, respectively (Figure 4b). Analysis of V-J pairing usage showed predominant pairing of TRBV6-2 with TRBJ2-5 (Figure 4c). Distribution analysis showed that approximately 30% of the top 100 TRB sequences expressed a specific TRB, suggesting expansion of specific T cells in the analyzed polyclonal population (Figure 4d). Saturation evaluation confirmed that the amount of sequencing data was reasonable, and that it accounted for most TCR sequences (Figure 4e).

## 4. Discussion

Clinical experience of the past decade has demonstrated that primary intraocular melanoma could be successfully managed by brachytherapy, which provides about an 80% five-year survival rate [19]. However, with time, nearly half of patients experience predominant liver metastases [2]. Despite recent advancements in immunotherapy for cutaneous melanoma, MUM remains intractable to this therapy, owing to the low frequency of UM-associated somatic mutations [20] and poor recruitment or quiescence of immune effectors to the hepatic MUM lesions [21]. The timing of UM metastasis to the liver is not well established, but it is believed that malignant cells migrate early and stay dormant as they adapt to the liver microenvironment. With the ultimate goal to target MUM cells and tumor seeds early and prevent MUM progression, in this study, we explored whether active vaccination could elicit UM-specific immunity. Previously, several attempts were made to target mutated tumor drivers (e.g., mutated BRAF, RAS) by immunotherapy. Some studies have shown that T cell response toward these proteins could be elicited by different vaccination approaches and enhanced by varying vaccine composition [22,23,24]. Using the flexibility of DNA vaccination platform that allows manipulation of vaccine structure and composition, we tested whether different vaccine designs could activate T cell-mediated immunity toward Q209L mutant GNAQ/GNA11, the most frequent tumor driver mutation in UM. GNAQ/GNA11 polypeptide sequence spanning the Q209L mutation site does not contain strong HLA-A*02:01 binders. Yet, we predicted that leucine, being a branched amino acid, stabilizes the HLA-A:02*01—peptide complex [25] and provides weak MHC-binding, making mutated peptide “visible” for the immune system. As naturally occurring mtGNAQ-specific T cells have not been isolated, it is plausible that mtGNAQ peptide–MHC complexes are not stable enough to produce strong T cell responses.

To improve the efficacy of mtGNAQ-specific vaccines, we employed several strategies including (i) VP22 and PADRE DNA vaccine fusion partners to enhance T cell activation, (ii) CCL21-enhanced DNA vaccination protocol [15], and (iii) introduction of HLA-A*02:01 anchoring substitutions to the putative immunogenic peptide [26,27,28,29]. Our data demonstrated that in-frame fusion of VP22 cDNA with antigen-encoding cDNA provided four times greater in vivo T cell activation than the construct lacking VP22 (Figure 1 and Figure 2). Despite prior research [13], the mechanisms of VP22-mediated immune response enhancement remain undefined [30]. Our in silico analysis showed that VP22 contains three regions enriched with human HLA-A*0101, -A*0201, and -A*0301 putative binding epitopes (Figure S4). It is plausible that the binding of these VP22 epitopes to MHC I increases clustering of the co-stimulatory molecules on the surface of vaccine-transduced DC and enhances the activation of tumor antigen-specific T cells. Although further investigation is required, our observations suggest that these regions can be independently used for the design of HLA-specific vaccines.

In the past, we have shown that the addition of CCL21 to vaccine composition enhances recruitment of CCR7^+^ T cells and mature DC to the vaccine administration site and facilitates T cell activation [15]. Introduction of anchoring amino acids into the antigenic epitope was successfully used in the past [26,27,28,29]. Our data showing that anchor-modified constructs (C4, C5) enhance UM-specific T cell immunity support this notion. Besides functional assays (IFNγ ELISpot, CTL and granzyme B activity), clonal expansion of vaccine-induced T cells was also supported by immuno-sequencing data showing the predominant expansion of specific T cell clones in a population of ex vivo activated T cells (Figure 4). Our in silico analysis also predicted that the Q209L mutation could create putative HLA-DR1, HLA-DR2, and HLA-DR5 binding epitopes (Table S2) that could be processed in an MHC II-dependent manner after lysis of the UM cells by vaccine-activated CTL and enhanced activation of T helper cells and antibody response [31]. These predictions have yet to be validated.

Our studies are limited to a rather small sample size used in the in vivo experimentation, yet the LaMorter power calculation on the obtained results (Figure 2d) showed that employed cohorts of animals provide 95% power to detect difference with alpha 0.01. This study is also limited by the lack of extended tumor monitoring in animals responding to vaccine by the inhibition of tumor progression. Yet, the timeframe was sufficient to compare different vaccine constructs for the ability to activate T cells and inhibit tumor progression. Due to the lack of immunocompetent animal models recapitulating human UM, this study is limited to the use of mouse melanocytic cells engineered to mimic human UM. Although artificial, this model allowed us to validate vaccine-mediated activation of mtGNAQ-specific immunity in vivo. Being a proof of concept, this study did not differentiate CD4- and CD8-mediated response, acquisition of immunologic memory, or durability of immune response. Our ex vivo findings provide proof-of-concept data demonstrating that optimization of the vaccine could enhance its efficacy. It is acknowledged that these data obtained with human leukocytes isolated from blood of a healthy donor may not be generalizable to UM patients. An important area for future studies will include further optimization of vaccine composition, broader investigation of the therapeutic capacity of vaccine-activated human T cells in animal models recapitulating human MUM, evaluation of ex vivo T cell activation protocol with UM patient-derived leukocytes, and testing of vaccine efficacy in humanized animal models.

## 5. Conclusions

Collectively, our findings demonstrate that mtGNAQ/GNA11 peptides harboring the Q209L substitution can be recognized by T cells activated by DNA vaccines in vivo and ex vivo. The data showed that anchoring modification(s) of the mtGNAQ epitope in the vaccine sequence could further enhance human T cell activity toward UM cells. These findings suggest that the established ex vivo protocol is suitable for activating UM-specific T cells suitable for MUM treatment. Although further studies with UM patient-derived cells are required to validate the production of functionally active T cells ex vivo, our data suggest that nucleic acid-based vaccination could be further improved to elicit protective immunity toward MUM.

## Data Availability

Data generated by the authors are included in this article. The raw data generated by the authors and T cell receptor (TCR) sequencing raw data files are available upon request.

## Supplementary Materials

The following supporting information can be downloaded at: https://www.mdpi.com/article/10.3390/cancers18030480/s1, Table S1. In silico analysis of GNAQ peptide binding to HLA-A* molecules. Table S2. In silico prediction of Q209L GNAQ peptide binding to HLA-DR* molecules. Figure S1. Alignment of mouse and human GNAQ sequences. Figure S2. Vaccine expression and development of mtGNAQ mouse cells. Figure S3. Ex vivo DC differentiation, vaccine expression, and real-time cytotoxicity. Figure S4. Analysis of VP22 putative MHC I-binding sites.

## Abbreviations

The following abbreviations are used in this manuscript:

UM	Uveal melanoma
MUM	Metastatic uveal melanoma
HLA	Human lymphocyte antigen
PADRE	Pan-DR-binding epitope
ICI	immune checkpoint inhibitors
PFS	Progression-free survival
OS	Overall survival
mt	Mutant
wt	Wild-type
DNA	Deoxyribonucleic acid
RNA	Ribonucleic acid
RT	Reverse transcription
VP22	Viral protein 22
TPA	12-O-tetradecanoylphorbol-13-acetate
CD	Cluster differentiation
DC	Dendritic cells
FACS	Fluorescence activated cell sorting
IFN	Interferon
ANOVA	analysis of variance
TCR	T cell receptor
CDR3	Complementarity-determining region 3
CCL21	-C-motif chemokine 21; Secondary lymphoid chemokine 21
CCR7	-C-motif chemokine receptor 7
ELISpot	Enzyme-Linked Immunosorbent Spot
CTL	Cytotoxic T lymphocyte
