# Supplementary material for: Cancer Vaccine Targeting Mutated GNAQ-Expressing Uveal Melanoma
Source: Cancers (Basel). 2026 Jan 31;18(3):480. doi: 10.3390/cancers18030480 (PMC12897428; doi:10.3390/cancers18030480)
Supplement: Supplementary file 1 [file cancers-18-00480-s001.zip › cancers-4066140-supplementary.pdf]

Supplementary Material S1

S1.1. Supplementary Materials

Cell cultures

Mouse melanocytic cells, Melan-a, and derivative cells expressing mtGNAQ and HLA-A2 were cultured in RPMI 1640 media supplemented with 10% fetal bovine serum (FBS), 2% L-glutamine, and beta mercaptoethanol. Primary uveal melanoma (UM) cells 92.1 and metastatic UM cell line UM002 were cultured in the RPMI1640 media supplemented with 10% FBS, 1% nonessential amino acid, 2% L-glutamine, 1% HEPES, 5000 IU penicillin, and 5000 µg/mL streptomycin. Human PBMC were isolated from HLA-A*02:01 donors' blood (StemCell Technologies, LeukoPak, Cat #70500.2) using SepMate-50 tubes (Cat #85450) with Lymphoprep density gradient (StemCell Technologies, Cat #07801). Monocytes and autologous T cells were isolated from PBMC using negative selection kits (StemCell Technologies, Cat #19359 and #17951, respectively). Isolation was performed according to manufacturer protocols. Dendritic cells were differentiated from monocytes and cultured in ImmunoCult-ACF dendritic cell medium using DC culture kit (StemCell Technologies, Cat #10985). T cells were cultured in ImmunoCult-XF T cell expansion medium (StemCell Technologies, Cat #10981) supplemented with human recombinant IL-2 (StemCell Technologies, Cat #78036.1). For T cell expansion, ImmunoCult human CD3/CD28 T cell activator (StemCell Technologies, Cat #10971) was used. All cells were maintained in a humidified atmosphere containing 5% CO_2 at 37°C.

Ex vivo T cell activation

For ex vivo activation of HLA-A*02:01-positive T cells with autologous DC, $CD14^+$ moncytes and pan-T cells were isolated from PBMC of healthy donors. Monocytes were differentiated into DC with DC differentiation kit (StemCell Technologies). Right after isolation, monocytes were placed in DC culture media supplemented with DC differentiation cocktail. After 3 days, this media was changed, and cells were cultured for 2 days. Immature DC were transduced with DNA vaccines via nucleofection using Nucleofector® II Device (Lonza) with DC nucleofection kit (Lonza, Cat #VPA-1004). After nucleofection, maturation cocktail was added and cells were exposed to autologous T cells at a ratio of 1:40 (DC:T). In 24 h, expression of CD80 and CD86 DC maturation markers was assessed by FACS using protein-specific antibodies (BioLegend, Cat #305414, #305207). Expression of the vaccine was evaluated by Western blot using GNAQ-specific antibodies (SantaCruz, Cat# SC-393). Re-activation of human T cells was performed under the same protocol.

IFNγ ELISpot assay

Mouse and human T cell activation was evaluated by IFNγ ELISpot assays using corresponding kits (eBioscience #88-7384-88; ImmunoSpot #hIFNg) as devised by the manufacturers. In these assays, mouse or human melanocytic cells expressing mutant or wild-type GNAQ were used as targets. Briefly, target cells were irradiated with 3,000 rad and washed with culture media. Cell density was adjusted to $4x10^5$ cells per ml. Then, 100 µl of target cell suspension were plated onto wells of ELISpot plates. For mouse and human cells, RPMI1640 medium supplemented with 10% FBS and CTL-Test medium (Immunospot, Cat #CTLT-005) were used, respectively. Mouse splenocytes and human T cells were resuspended in these media to $2x10^6$ cell per ml, and 100 µl of suspension were plated into ELISpot plates. Cells were incubated for 48h in a humidified atmosphere that contained 5% CO_2 at 37°C. Then, IFNγ-positive spots were visualized using reagents provided in respective kits as devised by the manufacturers. Wells were photographed and enumerated using AID vSpot plate reader (AID-diagnostika) with vSpot analysis software.

Cytotoxicity assay. To evaluate cytolytic activity of the ex vivo activated T cells, a FACS-based approach was used. Briefly, target cells were labeled with Vibrant DiO cell labeling dye at 1:2000 dilution for 10 min in serum-free media at 30^0C. After two washes, cells were resuspended in CTL-Test medium to $2x10^4$ cells per ml. Effector T cells were resuspended in the same medium to $2x10^6$ cells per ml. Then, 100 µl target cells and volumes of effector T cells corresponding to E:T ratios were mixed in U-bottom 96-well plates and incubated for 4 h at 37°C in a humidified atmosphere that contained 5% CO_2. After incubation, 5 µl of 7-ADD reagent (Invitrogen, Cat #A1310) were added to the wells to stain dead cells. Cells were incubated for 10 min and then evaluated by FACS using GuavaEasyCyte FACS system. DiO+ target cells were gated, and percentages of DiO+ 7-ADD+ cells were determined. Data were analyzed using GuavaSoft 2.7 software.

In vitro T cell aggregation assay.

Target cells (1x10^3 per well) expressing mutated and wild-type GNAQ were plated onto wells of a 6-well plate and allowed to proliferate for 3 days. Control and ex vivo activated T cells were labeled with Vibrant DiO dye by incubating 1x10^6 cells with 5 µl of the dye for 10 min. After washes, 1x10^3 T cells per well were added to wells with unlabeled targets. Cells were incubated for 1 h at 37^{0}C in a humidified atmosphere that contained 5% CO_2 in serum-free media, and accumulation of the fluorescently labeled T cells on targets were assessed by direct fluorescence using inverted fluorescent microscope (Nikon TS100) and Nikon D3-U camera and analyzed using NIS elements software (Nikon).

S1.2. Supplementary Tables

Table S1.

**Table S1.** ***In silico*** **analysis of GNAQ peptide binding to HLA-A* molecules**				
**GNAQ/GNA11**	**Sequence**	**Probability Score**	**Affinity (nM)**	**Binding**
**HLA-A*0101**				
Unmodified wild-type	FRMVDVGG**Q**	-24	28466.39	
Unmodified mutant	FRMVDVGG**L**	-22	20685.71	
Unmodified mutant	MVDVGG**L**RS	16	6387.89	WB
**HLA-A*0201**				
Unmodified wild-type	FRMVDVGG**Q**	-20		
Unmodified mutant	FRMVDVGG**L**	15	12589.43	WB
L209 at P6 with D205L and E212V	V<u>L</u>VGG**L**RS<u>V</u>	88	51.58	SB
L209 at P9 with R202L	F<u>L</u>MVDVGG**L**	64	11.07	SB
**HLA-A*0301**				
Unmodified wild-type	FRMVDVGG**Q**	-12	42549.94	
Unmodified mutant	FRMVDVGG**L**	-19	35449.75	
Unmodified wild-type	RMVDVGG**Q**R	-12	1400.04	
Unmodified mutant	RMVDVGG**L**R	5	460.77	WB
Prediction is based on binding and C-teminus cleavage model.<br>WB – weak binder; SB – Strong binder.				

Table S2.

**Table S2.** ***In silico*** **prediction of Q209L GNAQ peptide binding to HLA-DR* molecules.**		
HLA	Sequence	Binding probability
HLA-DR1	RMVDVGG**Q**R	10.1
HLA-DR1	RMVDVGG**L**R	13.8
HLA-DR2	RMVDVGG**Q**R	7.5
HLA-DR2	RMVDVGG**L**R	9.5
HLA-DR5	RMVDVGG**Q**R	6.0
HLA-DR5	RMVDVGG**L**R	14.0
Predicted HLA-DR binding threshold: 6.6		

Supplementary Material S2. Supplementary Figures

Figure S1

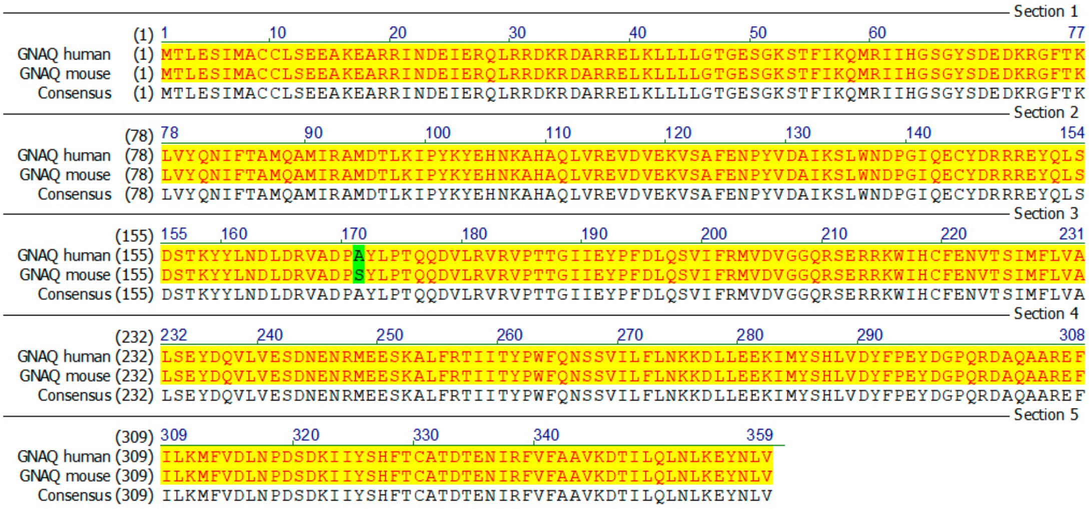

Figure S1. Alignment of mouse and human GNAQ sequences.

Alignment illustrates 99.9% homology between mouse and human GNAQ protein sequences with exception of the hydrophobic alanine (A171) in human GNAQ that changes to polar serine in the mouse protein.

Figure S2

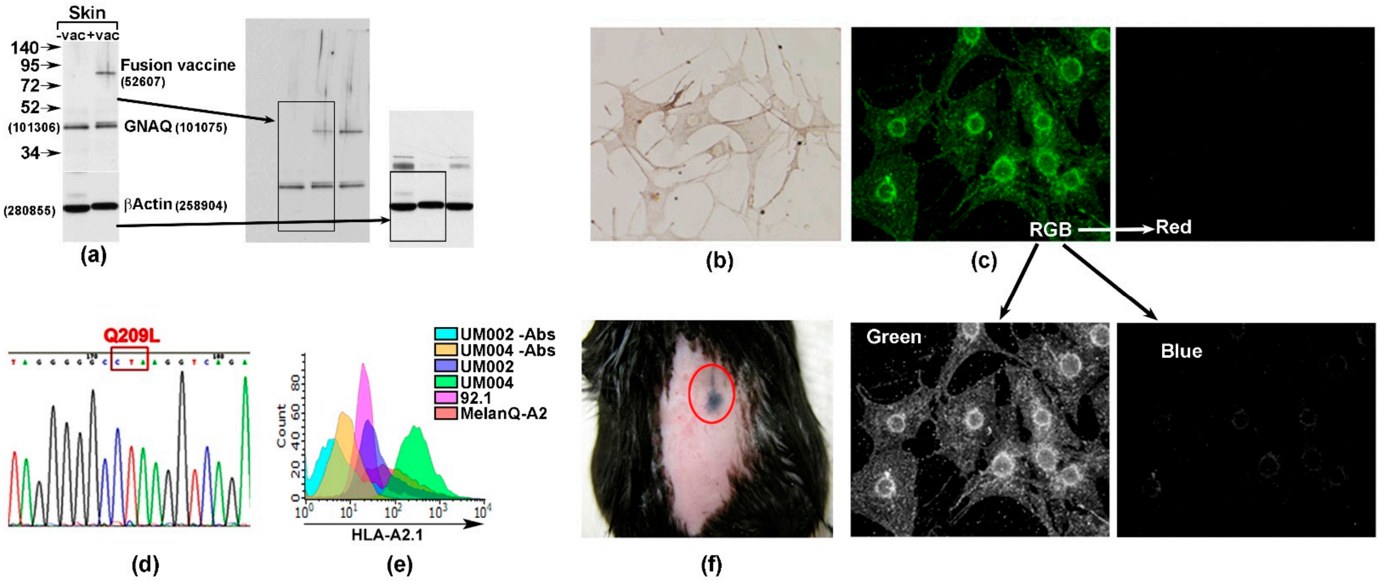

Figure S2. Vaccine expression and development of mtGNAQ mouse cells.

(**a**) Image of the Western blot illustrating expression of the fusion vaccine construct (C3) in mouse skin 48 h after ID EP. Mock EP skin lysates were used as control. Anti-mouse CCL21 antibodies are used to detect endogenous GNAQ (~42kDa) and fusion vaccine (~80 kDa) proteins, as indicated. βActin was used as loading control. (**b**, **c**) Micrographs illustrating morphology (**b**, light microscopy) and GNAQ (green) expression (**c,** indirect immunofluorescence) in mouse melanocytic cells (Melan-a) transduced with mtGNAQ and HLA-A2/K^b constructs. (**d**) Chromatogram showing predominant expression of CTA codon (indicated) encoding mutant L209. Total RNA from the clonal mtGNAQ-transduced and selected Melan-a cells was subjected to RT-PCR with GNAQ-specific primers. Generated products were subjected to Sanger sequencing. (**e**)FACS-based evaluation (chromatogram) of HLA-A*02 expression in uveal melanoma cells and generated MelanQ-A2 mouse cells expressing hybrid HLA-A2/Kb (indicated in the key). (**f**) Representative image of a mouse harboring blue nevus-like lesion (outlined) developed in the skin after intradermal injection of MelanQ-A2-expressing cells.

Figure S3

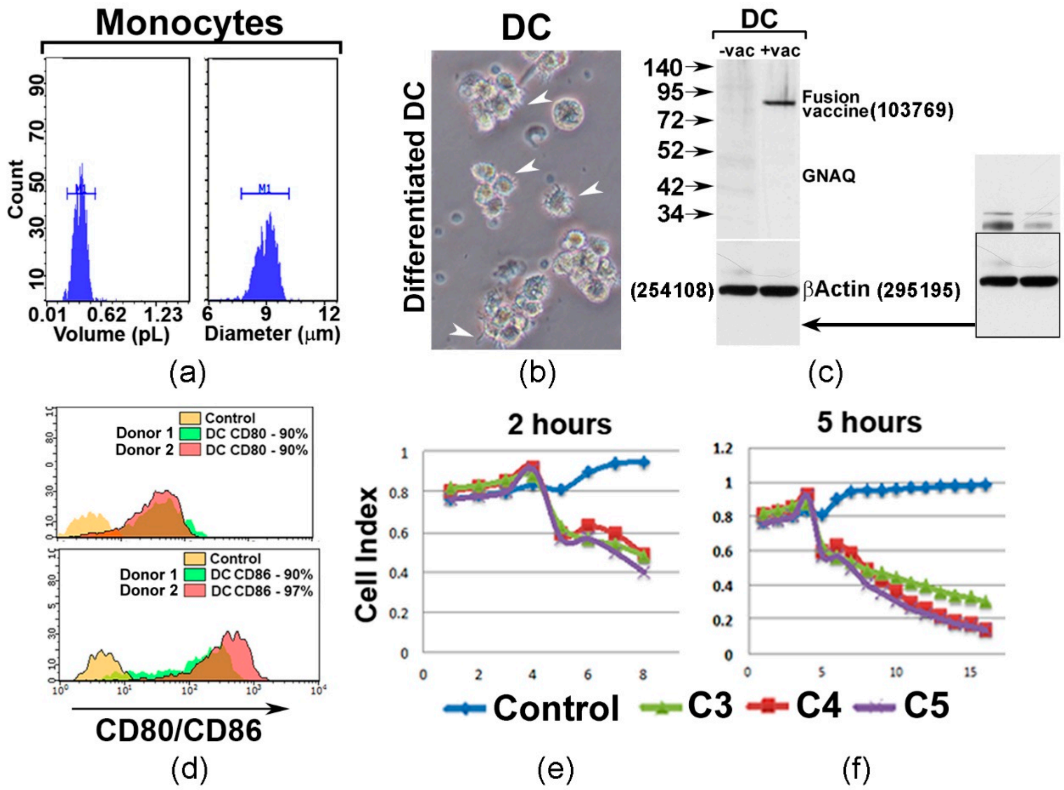

Figure S3. Ex vivo DC differentiation, vaccine expression, and real-time cytotoxicity.
(**a**) Negative selection provides homogeneous population of CD14+ monocytes. Volume and diameter are indicated. (**b**). Representative micrograph (light microscopy) illustrating ex vivo DC differentiation with noticeable dendrites (arrowheads). (**c**) Western blot assessment of fusion vaccine expression in ex vivo transduced DC. Fusion vaccine was detected with anti-mouse GNAQ antibodies. βActin was used as loading control. Molecular weight markers (kDa) are shown to the left of the panel. (**d**) Representative FACS profiles showing that more than 90% of differentiated DC expressed CD80 (top panel) and CD86 (bottom panel) maturation markers in two batches of differentiated DC (indicated in the key) after induction of maturation. Immature DC were used as controls. (**e**, **f**) Graphs illustrating xCELLigence real-time cytotoxicity assay with ex vivo activated T cells (indicated in the key) as effectors and 92.1 UM cells as targets at 40:1 ratio. Control—non-activated T cells. Cell index on y-axis depicts viability. Graphs show measurements at 2h (e) and 5 h (f) as indicated.

Figure S4

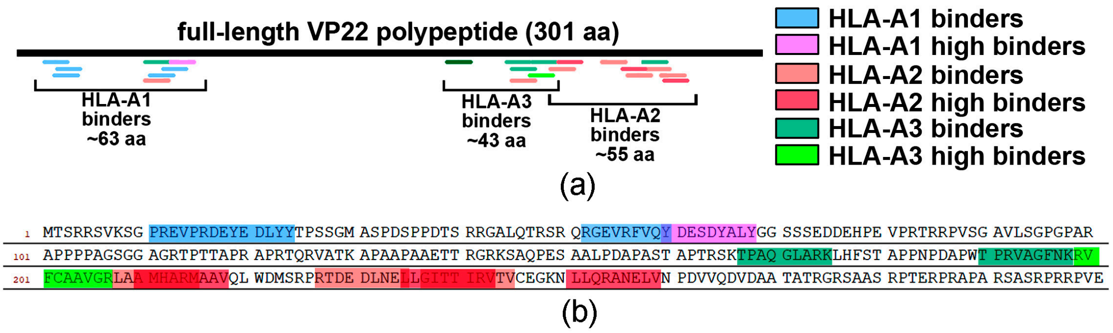

Figure S4. Analysis of VP22 putative MHC I-binding sites. (a) Schematic diagram of VP22 polypeptide depicting position of three regions enriched with HLA-A*01, -A*02, and A*03-putative binders predicted by in silico analysis. HLA binders are color-coded as indicated in the key. (**b**) VP22 amino acid sequence with selected HLA putative binders (highlighted as indicated in the key).
